# Couple‐Level Coping and Hope Interdependence in Hepatocellular Carcinoma After Transcatheter Arterial Chemoembolisation: Insights From an Actor–Partner Interdependence Model

**DOI:** 10.1155/jonm/3877436

**Published:** 2026-09-27

**Authors:** Yu Wei, Xiaofang Long, Ying Yin, Yan Li, Lei Sun, Ailing Hu, Yuan Liu

**Affiliations:** ^1^ Department of Cardiothoracic Surgery & Division of Vascular Interventional Radiology, The Third Affiliated Hospital of Sun Yat-Sen University, Guangzhou 510630, Guangdong, China, zssy.com.cn; ^2^ Department of Surgery, The Third Affiliated Hospital of Sun Yat-Sen University, Guangzhou 510630, Guangdong, China, zssy.com.cn; ^3^ Department of Surgery, The Third Affiliated Hospital of Sun Yat-Sen University Zhaoqing Hospital, Zhaoqing 526070, Guangdong, China; ^4^ Department of Gynecology, The Third Affiliated Hospital of Sun Yat-Sen University, Guangzhou 510630, Guangdong, China, zssy.com.cn; ^5^ Nursing Teaching & Research Office, The Third Affiliated Hospital of Sun Yat-Sen University, Guangzhou 510630, Guangdong, China, zssy.com.cn; ^6^ Nursing Department, The Sixth Affiliated Hospital of Sun Yat-Sen University, Guangzhou 510655, Guangdong, China, sysu.edu.cn; ^7^ Biomedical Innovation Centre, The Sixth Affiliated Hospital of Sun Yat-Sen University, Guangzhou 510655, Guangdong, China, sysu.edu.cn

**Keywords:** actor–partner interdependence model, dyadic coping, hepatocellular carcinoma, hope, spouses, transcatheter arterial chemoembolisation

## Abstract

**Background:**

Hepatocellular carcinoma (HCC) and transcatheter arterial chemoembolisation (TACE) are physically demanding for patients and their caregivers, as well as psychologically stressful. Spouses are often the main informal caregivers, so illness‐related stress after TACE is experienced by the couple. Hope can provide psychological support for people with cancer, and dyadic coping refers to how couples cope with the stress of illness through communication. However, evidence remains limited regarding actor and partner associations between hope and dyadic coping in HCC patient–spouse dyads after TACE.

**Aim:**

To examine the actor and partner associations between hope and dyadic coping in HCC patient–spouse dyads after TACE using the actor–partner interdependence model (APIM).

**Methods:**

This cross‐sectional study included 177 patient–spouse dyads recruited from a tertiary hospital in China between October 2023 and June 2024. Participants completed questionnaires on hope and dyadic coping. Data were analysed using SPSS 25.0 and AMOS 24.0. Distinguishable dyads were specified in the APIM, controlling for patient age, Child–Pugh grade, and number of TACE sessions.

**Results:**

Both patients and their spouses reported moderate dyadic coping and relatively high hope. Hope was positively correlated with dyadic coping within and between partners. APIM revealed significant actor associations, indicating that higher hope was associated with better individual dyadic coping in patients (B’ = 0.289, 95% CI [0.109, 0.451], *p* < 0.001) and spouses (B’ = 0.381, 95% CI [0.221, 0.521], *p* < 0.001). Significant partner associations were also observed from patients to spouses (B’ = 0.160, 95% CI [0.001, 0.308], *p* < 0.05) and from spouses to patients (B’ = 0.181, 95% CI [0.031, 0.322], *p* < 0.05). These findings suggest that hope is associated with dyadic coping in both patients and spouses, underscoring the interdependent nature of psychological adjustment among couples managing HCC after TACE.

**Conclusions:**

Hope was associated with dyadic coping in both patients and spouses, highlighting the interdependent nature of psychological adjustment in couples coping with HCC after TACE.

**Implications for Nursing Management:**

Nurse managers should recognise HCC patient–spouse dyads as an interdependent unit and incorporate assessment of both partners’ hope and dyadic coping into oncology care management.

## 1. Introduction

Hepatocellular carcinoma (HCC) is one of the most common malignancies worldwide and remains a leading cause of cancer‐related death, particularly in regions with a high burden of chronic hepatitis and liver cirrhosis [1, 2]. Transcatheter arterial chemoembolisation (TACE) is widely used for patients with unresectable or intermediate‐stage HCC and plays an important role in tumour control and survival prolongation [3, 4]. However, TACE is also associated with considerable physical and psychological burden. Following TACE, patients may experience postembolisation symptoms, including pain, fever, nausea, fatigue, appetite loss and liver function deterioration, as well as uncertainty regarding tumour response, recurrence and prognosis [4, 5]. These treatment‐related and disease‐related stressors may undermine patients’ emotional adjustment, treatment confidence and daily functioning [6].

The impact of HCC and TACE extends beyond patients themselves. Spouses are often the primary informal caregivers and play a central role in treatment decision‐making, symptom monitoring, daily care and emotional support [7]. Evidence from Asian countries indicates that cancer‐related health and adaptation may be interdependent between patients and family caregivers [8, 9]. For instance, a study from South Korea demonstrated that the physical and mental health of patients with colorectal cancer and their family caregivers were significantly interdependent [8]. Consequently, spouses may also experience considerable caregiving burden, emotional distress, role strain and uncertainty about the future [7, 10]. Cancer and its treatment should, therefore, be viewed as a shared stressor for patient–spouse dyads rather than an isolated individual experience. Consistent with this view, cancer has been conceptualised as a “we‐disease” that affects both members of the couple and requires joint adaptation [11]. Moreover, a study from Thailand found that family involvement was associated with improved psychological outcomes in patients with advanced HCC, highlighting the importance of family‐centred supportive care in Asian oncology settings [12]. Understanding how patients and spouses draw on psychological and relational resources is, therefore, essential for optimising supportive care and nursing management.

The systemic transactional model of dyadic coping provides a useful theoretical framework for understanding couple adaptation to illness‐related stress [13, 14]. According to this model, stress is not merely experienced within individuals but is communicated, appraised and managed within the couple system. One partner’s stress experience and coping responses may be associated with the other partner’s emotional state, support behaviours and coping strategies [13, 14]. Dyadic coping refers to the process through which partners communicate stress, provide emotional or practical support and engage in joint efforts to manage shared stressors [13]. In the context of cancer, effective dyadic coping may help couples maintain emotional connection, share illness‐related responsibilities, reduce psychological distress and adapt to treatment demands [11, 15]. Therefore, dyadic coping is particularly relevant for patients with HCC after TACE and their spouses, who must jointly manage disease uncertainty, repeated treatment cycles, posttreatment symptoms and caregiving challenges [16, 17].

Hope is another important psychological resource in cancer adaptation. Hope has been conceptualised as an individual’s perceived capacity to identify pathways towards meaningful goals and sustain the agency needed to pursue those goals [18]. The Herth hope framework further emphasises temporality and future orientation, positive readiness and expectancy and interconnectedness with self and others [19]. For patients with cancer, hope may help maintain meaning, treatment motivation and psychological resilience despite uncertainty and symptom burden [18, 20, 21]. For spouses, hope may buffer caregiving stress, support sustained engagement in care and facilitate positive expectations about the future [22]. However, hope has often been examined as an individual psychological attribute, while its interpersonal implications within patient–spouse dyads remain insufficiently understood.

The integrated framework combining Snyder’s hope theory [18] and Bodenmann’s systemic transactional model [13, 14] of dyadic coping serves as the core theoretical framework of the present study. This dual‐theory integration delivers a coherent theoretical rationale to unpack the bidirectional interdependent associations between hope and dyadic coping at both intrapersonal (actor) and interpersonal (partner) levels and establishes rigorous theoretical underpinnings for subsequent actor–partner interdependence model (APIM) statistical analyses [23, 24].

Theoretically, hope may be closely related to dyadic coping. Individuals with higher levels of hope are more likely to perceive stressful situations as manageable, identify possible pathways to address difficulties and maintain motivation to pursue valued goals [18]. In a cancer context, hopeful patients or spouses may be more willing to communicate illness‐related concerns, seek and provide support, participate in joint problem‐solving and maintain constructive interactions during treatment [25]. Thus, hope may enhance an individual’s own dyadic coping. This association represents an actor association, a concept well‐established in dyadic cancer research [23, 26], whereby a person’s hope is presumed to be linked to their own coping within the couple relationship.

In addition, hope may have interpersonal associations within patient–spouse dyads. Emotional states, expectations and coping orientations can be transmitted between partners through communication, behavioural modelling, reciprocal support and the overall relational climate [11, 13, 14, 27]. A patient with higher hope may reduce the spouse’s sense of helplessness and encourage the spouse to engage in supportive and collaborative coping. Similarly, a hopeful spouse may provide reassurance, emotional stability and future‐oriented encouragement, which may facilitate the patient’s active engagement in dyadic coping. Hope may, therefore, function not only as an individual psychological resource but also as an interpersonal resource that supports the partner’s coping. This association represents a partner relationship, whereby one individual’s hope is associated with the other partner’s dyadic coping [16, 23].

Although previous studies have examined hope, coping, psychological distress or caregiver burden among cancer populations, several knowledge gaps remain. First, most studies have focused on patients or caregivers separately and have not sufficiently considered the interdependence between patients and spouses [28, 29]. Second, existing dyadic coping research has been conducted mainly in other cancer populations, whereas dyadic evidence specifically in HCC patient–spouse dyads after TACE remains limited, with only a few recent studies beginning to address this population [16, 17, 30].

These couples may face distinctive challenges arising from chronic liver disease, postembolisation symptoms, repeated treatment cycles and uncertainty regarding tumour response. Third, traditional individual‐level statistical approaches often fail to account for the nonindependence of dyadic data and cannot determine whether an individual’s dyadic coping is associated with their own psychological resources, their partner’s resources or both [23, 31].

From a nursing management perspective, identifying dyadic psychological resources can help nurse managers design couple‐centred assessment and intervention pathways [32, 33]. Routine screening of both patients’ and spouses’ hope and dyadic coping may allow oncology nurses to identify dyads at risk of poor adjustment and provide timely psychosocial support [32, 34]. Such an approach is consistent with the shift from patient‐centred to family‐ and dyad‐centred cancer care, in which both members of the couple are recognised as active participants in illness management and supportive care [7, 12, 32, 33]. Evidence regarding actor and partner associations between hope and dyadic coping may, therefore, provide a theoretical and empirical basis for developing hope‐centred dyadic nursing interventions for patients with HCC after TACE and their spouses.

The APIM is well suited to address these gaps. As a statistical model for dyadic data analysis, APIM allows simultaneous estimation of actor associations and partner associations while accounting for the nonindependence between members of a dyad [16, 23]. Applying APIM to HCC patient–spouse dyads after TACE can clarify whether hope is associated only with one’s own dyadic coping or also with one’s partner’s dyadic coping. Such evidence may help nursing managers and clinical nursing teams develop dyad‐centred assessment, education, communication guidance and supportive care strategies [13, 16].

The present study should also be distinguished from prior work on psychological distress among spouses of patients with familial clustering of hepatitis B virus–related HCC [35]. Although both studies involve HCC and spouses, the current study examines hope and dyadic coping in patients after TACE and their spouses using the APIM, whereas the prior study focused on psychological distress and related factors in spouses of patients with familial HCC. There is no overlap in authors, institutions, participants, datasets or prior dissemination between the two studies.

Accordingly, this study aimed to describe hope and dyadic coping and examine their actor and partner associations among patients with HCC after TACE and their spouses. Specifically, the objectives were to: (1) assess levels of hope and dyadic coping in patients and spouses, (2) examine correlations among patients’ and spouses’ hope and dyadic coping and (3) estimate actor and partner associations between hope and dyadic coping using APIM.

Two hypotheses were proposed:

 Hypothesis 1: An individual’s hope will be positively associated with their own dyadic coping, representing a positive actor effect.
 Hypothesis 2: An individual’s hope will be positively associated with their partner’s dyadic coping, representing a positive partner effect.

Given the cross‐sectional design, these hypotheses concern statistical associations rather than temporal or causal relationships.

## 2. Methods

### 2.1. Research Design

This study employed a correlational, cross‐sectional design to examine the dyadic relationships between hope levels and dyadic coping in HCC patients after TACE and their spouses. Data were collected at a single time point and analysed using the APIM to estimate actor and partner associations within the couple dyad. Given the cross‐sectional nature of this design, pretreatment baseline data were not available, and thus pre‐ versus posttreatment comparisons could not be performed. The study instead focused on characterising the posttreatment status of dyadic coping and hope, as well as their interdependent associations, to provide preliminary evidence for future longitudinal investigations.

### 2.2. Participants and Sampling

An a priori power analysis was conducted using APIMPowerR to estimate the minimum sample size required for the present study. Statistical power was set at 0.80. The expected effect sizes were specified as 0.30 for the actor effect in patients, 0.35 for the actor effect in spouses, 0.22 for the partner effect in patients and 0.22 for the partner effect in spouses. The correlation between predictor variables was set at 0.40, and the correlation between residual errors was set at 0.50. Based on these assumptions, the analysis indicated that a minimum of 162 dyads was required. Therefore, the final sample of 177 patient–spouse dyads was considered adequate for the APIM analysis.

Convenience sampling was used to recruit 177 patient–spouse dyads from a tertiary‐level hospital in Guangzhou, China, between October 2023 and June 2024. The inclusion criteria for patients were as follows: (1) had a clinical diagnosis of HCC, (2) were aged ≥ 22 years for men and ≥ 20 years for women, (3) were married and had a spouse who was the primary caregiver, (4) had undergone at least one TACE procedure and (5) provided informed consent. Patients with psychiatric or cognitive disorders, communication difficulties, severe organic diseases (e.g., heart, liver or renal failure) or other malignancies were excluded. Spouses were eligible if they were aged ≥ 22 for men and ≥ 20 for women, provided informed consent and did not have psychiatric, cognitive or communication disorders.

According to the Marriage Law of the People’s Republic of China, the legal minimum ages for marriage are 22 for men and 20 for women. As all participants met these age requirements and those with cognitive impairments were excluded, all enrolled couples were legally married and deemed capable of providing informed consent.

All patient participants had primary HCC and had undergone at least one TACE procedure. Among these patients, 86.4% had not undergone surgical treatment, whereas 13.6% had previously undergone hepatic resection and subsequently received TACE because of postoperative recurrence or as adjuvant therapy.

### 2.3. Scale Assessment

#### 2.3.1. General Information Survey

For this investigation, a general information questionnaire created by the researcher was employed. It collected sociodemographic data from both patients and spouses, encompassing gender, years of age, attained education, employment status, household income and place of residence. Disease‐related information was collected from patients only and included time since diagnosis, Child–Pugh class, the number of prior TACE sessions, the number of previous local ablation treatments for liver cancer, whether the patient had undergone surgical resection and whether targeted therapy, immunotherapy or other adjuvant treatments had been received.

#### 2.3.2. Dyadic Coping Inventory (DCI)

The Chinese version of the DCI was used to assess dyadic coping for this study [36]. This inventory evaluates the stress communication quality and the coping strategies within a couple when facing stressful situations. The psychometric performance of the DCI in this investigation was substantiated by excellent internal consistency, with Cronbach’s *α* coefficients of 0.919 for patients and 0.913 for their spouses.

The 37 items of the DCI are organised into six subscales: supportive coping, delegated coping, common coping, negative coping, stress communication and coping quality evaluation. All items are on a 5‐point Likert scale, and the options are ‘*very rarely*’ (1) and ‘*very often*’ (5). The coping quality subscale is scored separately and not included in the total score. A higher total score indicates that people engage in more frequent positive coping behaviours and have a generally stronger dyadic coping ability.

#### 2.3.3. Herth Hope Index (HHI)

The Chinese version of the HHI was employed to assess participants’ hope levels [19]. In this study, the HHI demonstrated good internal consistency, with Cronbach’s *α* coefficients of 0.833 for patients and 0.853 for their spouses. Twelve components total, organised into three dimensions, make up the HHI. Stronger overall scores indicate a stronger degree of hope. The Likert scale has four points, with 1 reflecting ‘*strongly disagree*’ and 4 reflecting ‘*strongly agree*’. Three levels of the total score can be distinguished: low (scores 12–23), moderate (24–35) or high (36–48).

### 2.4. Data Collection

Data were collected during the patients’ hospitalisation for TACE. Patients and their spouses completed the questionnaires on the day before the patient’s discharge, such that hope and dyadic coping were assessed during the early post‐TACE inpatient period. After providing informed consent, each member of the dyad completed the questionnaires independently and in private to minimise mutual influence. Researchers were available to answer questions and clarify questionnaire items when needed. All questionnaires were checked for completeness and collected immediately after completion.

A total of 192 dyadic questionnaire sets were distributed. Of these, 177 complete and eligible dyadic sets were retained for analysis, yielding a valid response rate of 92.19%. Incomplete questionnaires were excluded from the analysis. Specifically, a dyadic set was excluded if either member of the couple failed to complete the questionnaire, if a participant showed an apparent straight‐line response pattern and declined to verify or revise the responses, or if substantial missing data were present. Missing data were handled using complete‐case analysis, such that only dyads with complete data on all variables included in the analysis were retained.

### 2.5. Ethical Considerations

The hospital’s Medical Ethics Committee formally approved this study on October 30, 2023 (Approval No.: ZDFSYLLII2023‐267‐01). All prospective volunteers were informed by the researchers of the reasons for the study before signing up. Written informed consent was obtained from all subjects before the commencement of the study. To protect the privacy of the participants, an anonymous ID number was used to collect all the data, and no personally identifiable information was disclosed outside. If the participant experienced any discomfort during the completion of the questionnaire, we would stop at once and offer other help. All participants were informed clearly that at any time during the study, they could choose to withdraw from the research, and this would not affect their regular medical care or change the plan for continuous treatment.

### 2.6. Reporting Method

This study was reported in accordance with the Strengthening the Reporting of Observational Studies in Epidemiology (STROBE) guidelines for cross‐sectional studies. The completed STROBE checklist is provided as supporting information available (here).

### 2.7. Data Analysis

IBM SPSS Statistics 25.0 was used for data analysis. Frequencies and percentages were used to present categorical data, and means and standard deviations were employed for continuous data; all analyses were performed in IBM SPSS Statistics 25.0. Categorical variables were presented as frequencies and percentages, and continuous variables were shown as means and standard deviations. Structural equation modelling was performed using IBM SPSS Amos 24.0. The distribution of continuous variables was assessed using the Kolmogorov–Smirnov test, visual inspection of histograms and P–P plots, and skewness and kurtosis values. Although the hope and dyadic coping scores were not normally distributed, they were presented as means and standard deviations in the descriptive tables to maintain consistency with previous studies.

Between‐group differences were examined using independent‐samples *t* tests, and within‐dyad differences in hope and dyadic coping were examined using paired‐samples *t* tests. Although some variables showed departures from normality, paired *t* tests were retained because the sample size was relatively large (177 dyads), and the test is generally robust to moderate nonnormality. Pearson’s correlation coefficients were calculated to assess associations between hope and dyadic coping. Missing data were handled using complete‐case analysis; dyads with missing data on any variable included in the analyses were excluded.

The APIM was estimated using structural equation modelling in AMOS. Because patients with HCC receiving TACE and their spouses were distinguishable by illness status, the standard APIM for distinguishable dyads was first tested using a saturated model. Equality constraints were then imposed on the actor effects and partner effects to examine whether the dyads could be treated as indistinguishable. Maximum likelihood estimation was used to estimate model parameters. To account for potential nonnormality, bootstrap resampling with 5000 samples was performed, and bias‐corrected 95% confidence intervals were calculated. Model fit was evaluated using the *χ* ^2^/df ratio, goodness‐of‐fit index (GFI), adjusted goodness‐of‐fit index (AGFI), normed fit index (NFI), comparative fit index (CFI) and root mean square error of approximation (RMSEA) with its 95% confidence interval. Patient age, Child–Pugh grade, and number of TACE sessions were included as covariates in the APIM.

Based on the saturated model, if the absolute values of both actor effects exceeded 0.10, the *k* parameter was estimated using an APIM with phantom variables. A bootstrap procedure with 5000 resamples was used to obtain the 95% confidence intervals for the k values. These results were then used to determine the dyadic pattern, which was further examined by constraining the k values. All statistical tests were two‐tailed, and *p* < 0.05 was considered statistically significant.

## 3. Results

### 3.1. Clinical and Sociodemographic Features of the Participants

The sample comprised 177 dyads of patients with HCC who had undergone TACE and their spouses. Among the patients, 154 (87.01%) were male and 23 (12.99%) were female, with a mean age of 52.89 years (SD = 9.86; range, 29–82). Regarding education, 88 patients had an education level below junior high school, 51 had completed senior high school and 38 had attained junior college education or above. A total of 106 patients (59.89%) lived in urban areas and 71 (40.11%) in rural areas. Most families (133, 75.14%) reported a monthly per capita income of < *¥*5000 CNY, whereas 44 (24.86%) reported an income of ≥ ¥5000 CNY. For 87.01% of patients, hospitalisation expenses were partially or fully reimbursed, whereas 12.99% paid entirely out of pocket.

The mean time of diagnosis was 16.05 months (SD 26.92). Among those with a history of treatment, 13.56% had previously undergone hepatic resection followed by TACE, and 86.44% had received TACE without prior surgery. At the time of assessment, 73.45% of the patients were Child–Pugh Grade A, and 26.55% were Grades B or higher. Among the TACE sessions, 42.94% had received one, 44.63% had received two to four and 12.43% had received five or more. The extra treatments were not mutually exclusive; 16.95% had received local ablative therapy, 52.54% targeted therapy, 40.68% immunotherapy and 57.63% other treatments (such as radioactive seed implantation or hepatic arterial infusion chemotherapy). The spouse subgroup comprised 154 women (87.01%) and 23 men (12.99%), with a mean age of 50.98 years (SD 10.03; range 29–77).

### 3.2. Comparisons of Dyadic Coping and Hope Between Patients and Spouses

The total dyadic coping score was 126.93 (SD = 17.32) in patients after TACE, while it was 125.25 (SD = 17.30) in spouses, both indicating a moderate level. The total hope score was 39.11 (SD = 3.97) in patients and 38.76 (SD = 4.26) in spouses, suggesting a relatively high level for both groups. Table 1 presents the detailed results.

**TABLE 1:** Comparison of dyadic coping and hope levels between patients with HCC after TACE and their spouses (*N* = 177 dyads).

Variables	Patients (*M* ± SD)	Spouses (*M* ± SD)	*t*	*p*
Dyadic coping	126.93 ± 17.32	125.25 ± 17.30	1.430	0.155
Negative dyadic coping	30.97 ± 6.74	30.86 ± 6.55	0.228	0.820
Positive dyadic coping	95.95 ± 16.06	94.39 ± 16.12	1.406	0.162
Stress communication	27.79 ± 5.28	26.96 ± 5.22	2.199	0.029^∗^
Delegated dyadic coping	14.58 ± 2.71	14.37 ± 2.49	1.023	0.308
Supportive dyadic coping	35.75 ± 6.42	35.34 ± 6.43	0.867	0.387
Common dyadic coping	17.84 ± 3.44	17.71 ± 3.72	0.481	0.631
Hope	39.11 ± 3.97	38.76 ± 4.26	1.022	0.308
Positive outlook on the future	12.79 ± 1.54	12.82 ± 1.68	0.284	0.776
Taking positive actions	13.14 ± 1.54	13.16 ± 1.54	0.159	0.874
Maintaining close relationships	13.07 ± 1.52	12.76 ± 1.51	2.454	0.015^∗^

^∗^ *p* < 0.05.

### 3.3. Correlations Between Dyadic Coping and Hope

Significantly positive relationships were found using Pearson’s correlation analysis between patients’ dyadic coping and their own hope levels (*p* < 0.01), as well as with their spouses’ hope levels (*p* < 0.01). Correspondingly, hope levels in spouses demonstrated significant positive associations not only with their own dyadic coping (*p* < 0.01) but also with that of the patients (*p* < 0.01). Interpartner analyses further revealed statistically significant correlations between patients’ and spouses’ dyadic coping measures (*p* < 0.01), as well as between their respective hope levels (*p* < 0.01). Table 2 displays the intercorrelations among the study variables.

**TABLE 2:** Correlations between dyadic coping and hope levels in HCC patients after TACE and their spouses (*N* = 177 dyads).

Variables	1	2	3	4
1. Patient dyadic coping	—			
2. Spouse dyadic coping	0.596^∗∗^	—		
3. Patient hope level	0.363^∗∗^	0.309^∗∗^	—	
4. Spouse hope level	0.302^∗∗^	0.441^∗∗^	0.388^∗∗^	—

^∗∗^ *p* < 0.01.

### 3.4. APIM of Dyadic Coping and Hope

Hope was specified as the predictor and dyadic coping as the outcome in a distinguishable dyadic APIM, with patient age, Child–Pugh grade, and number of TACE sessions included as covariates. All fit indices and parameter estimates reported below were derived from the distinguishable APIM without equality constraints on the actor and partner effects, estimated using maximum likelihood. To achieve model identification, partial zero constraints were imposed on the covariances among the exogenous predictors, retaining only the covariance between patient hope and partner hope. After adjustment for these covariates, the model demonstrated good fit to the data (*χ* ^2^/df = 0.541, GFI = 0.992, AGFI = 0.975, NFI = 0.972, CFI = 1.000 and RMSEA < 0.001, 95% CI [0.000, 0.048]). The adjusted R^2^ values indicated that the model explained 0.167 of the variance in patient dyadic coping and 0.203 of the variance in spouse dyadic coping. In addition, none of the three covariates were significantly associated with either patients’ or spouses’ total dyadic coping scores.

Significant actor associations were observed for both members of the dyad: Patients’ hope was positively associated with their own dyadic coping (B’ = 0.289, *t* = 3.918 and *p* < 0.001), and spouses’ hope was positively associated with their own dyadic coping (B’ = 0.381, *t* = 5.252 and *p* < 0.001). Significant partner associations were also identified: Patients’ hope was positively associated with spouses’ dyadic coping (B’ = 0.160, *t* = 2.218 and *p* = 0.027), and spouses’ hope was positively associated with patients’ dyadic coping (B’ = 0.181, *t* = 2.427 and *p* = 0.015). These findings indicate that dyadic coping was associated with both individuals’ own hope and their partners’ hope.

Based on the saturated model, the absolute standardised actor associations for both patients and spouses exceeded 0.10; therefore, *k* values were estimated using an APIM with phantom variables. Bootstrap resampling with 5000 iterations yielded a *k* value of 0.581 (95% CI [0.088, 2.447]) for patients and 0.450 (95% CI [0.011, 1.310]) for spouses. These results suggest that the actor associations were approximately twice as large as the partner associations, indicating a mixed pattern for both patients and spouses. This dyadic pattern was further supported by the constrained model. Overall, in this distinguishable dyadic relationship, both patients’ and spouses’ dyadic coping were affected not only by their own hope levels but also by their partners’ hope levels. Detailed parameter estimates are presented in Table 3, and the final model is shown in Figure 1.

**TABLE 3:** Actor and partner effects of hope on dyadic coping: an APIM analysis (*N* = 177 dyads).

Path	B	SE	B′	95% CI	*t*	*p*
Actor effects						
Patient hope ⟶ Patient dyadic coping	1.260	0.322	0.289	0.109, 0.451	3.918	< 0.001
Spouse hope ⟶ Spouse dyadic coping	1.548	0.295	0.381	0.221, 0.521	5.252	< 0.001
Partner effects						
Patient hope ⟶ Spouse dyadic coping	0.697	0.314	0.160	0.001, 0.308	2.218	0.027
Spouse hope ⟶ Patient dyadic coping	0.732	0.302	0.181	0.031, 0.322	2.427	0.015

*Note:* Estimates shown in Table 3 were derived from the distinguishable APIM with partial zero constraints imposed on the covariances among exogenous predictors, estimated using maximum likelihood and adjusted for patient age, Child–Pugh grade and number of TACE sessions.

**FIGURE 1:**
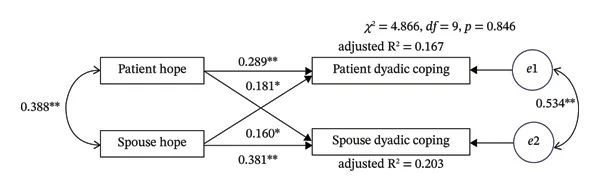
The APIM of hope and dyadic coping in HCC patients after TACE and their spouses. Note: Controlling for patient age, Child–Pugh grade and number of TACE sessions. ^∗^ *p* < 0.05 and ^∗∗^ *p* < 0.01.

## 4. Discussion

### 4.1. Dyadic Coping Levels Are Moderate Among HCC Patients After TACE and Their Spouses

The present study found that both patients with HCC after TACE and their spouses reported moderate levels of dyadic coping, which is consistent with the findings of Zhang et al. [37] in couples undergoing haematopoietic stem cell transplantation. This suggests that couples had some capacity to cope with illness‐related stress together, although their dyadic coping still has room for improvement.

Patients undergoing TACE are often in the intermediate or advanced stages of disease, with a complex clinical course and a high risk of recurrence. Repeated TACE sessions are frequently required, and 60%–80% of patients may experience postembolisation syndrome, including fever, abdominal pain, nausea, and vomiting [38]. The above treatment‐related symptoms may cause physical discomfort and an increased burden of caregiving for the couple, as well as psychological stress for themselves; thus, their joint coping capacity is reduced. Patients scored significantly higher than their spouses on the stress communication dimension, a finding consistent with previous reports by Zhang [37] and Rottmann [39]. A probable reason is that, as the direct subjects of treatment, patients may be more inclined to report illnesses or other anxieties; at the same time, spouses are less likely to express their own stress and emotions. In this study, most spouses were female. Female caregivers often bore the main responsibility for caregiving and housework and were under financial and emotional pressure. For these reasons, spouses may be reluctant or unable to talk about their stress openly. Cultural context may also explain the above results. Compared with the results in some Western countries, couples in many Asian cultural contexts are more likely to have a reserved coping style and rely more heavily on protective buffering [40]. Spouses in traditional Chinese culture may prefer to conceal their suffering and emotional distress out of fear of bothering the patient. The above tendency may reduce open‐stress communication in the dyad and partially explain the lower scores in stress communication for spouses.

### 4.2. Hope Levels Were Relatively High in Patients With HCC After TACE and Their Spouses

Both the patients and their spouses reported a relatively high level of hope in this study, and this was higher than what had been recorded in previous research by Li et al. [41], Wang et al. [25] and Yang et al. [42]. In short, all of the above results indicate that most couples in this sample were in a relatively favourable psychological state despite the problems of HCC and TACE treatment.

The reasons for the above differences may be variations in the social and demographic characteristics of the sample. Most of the patients in this study were city dwellers residing near the hospital and other community institutions. With the continuous development of China’s healthcare system, more and more people are covered by all sorts of insurance, and thus the economic burden on the families of those affected will be relatively light. Previous studies have shown that good social support can increase people’s sense of hope in life for those with long‐term illnesses [21, 25]. In addition, the patients scored significantly higher than their spouses on the item about close interpersonal relationships. Based on the above, it can be concluded that people will feel more hopeful when in touch with their families, friends and others. Spouses are more likely to take on the burden of caregiving and meet the demands of sick relatives, neglecting their own emotional needs and social lives [43].

Hope serves as a source of emotional support for individuals with cancer. A high sense of hope is related to more adaptive coping behaviour, better psychological health and a strong will‐to‐live in the face of illness [20, 44]. Fostering hope is particularly important for patients undergoing multiple TACE sessions, given that treatment can be prolonged and outcomes often remain uncertain. Hope can help patients and their families continue to participate in treatment and daily life despite the difficulties caused by a long illness [45, 46]. At the same time, hope should be grounded in clinical reality. In patients with serious illness, expectations shaped by unrealistic hope may cause substantial psychological distress if treatment does not progress as expected [47]. Accordingly, nursing interventions should aim not only to foster hope in patients and their spouses but also to help them cultivate realistic, flexible and sustainable hopefulness throughout the treatment trajectory.

### 4.3. Actor and Partner Associations Between Hope and Dyadic Coping

The present study revealed a positive correlation between hope levels reported by patients with HCC after TACE and those reported by their spouses. This finding aligns with prior evidence that couples may share and reinforce psychological resources when facing cancer‐related stress [25].

In addition, some scholars have studied the association of hope and dyadic coping together. According to the findings on actors, greater hope was associated with more effective dyadic coping for both patients and their spouses. In other words, a positive hope for one’s own coping was positively associated with the couple’s ability to cope well together. In short, the above results are in line with Hypothesis 1 and support Snyder’s hope theory [18], which considers hope to be a combination of agency thinking and pathways thinking. Agency thinking reflects an individual’s motivation to pursue goals, whereas pathways thinking refers to the perceived ability to identify workable routes towards those goals. In the context of HCC and repeated TACE, individuals with higher hope may be more likely to maintain motivation and identify practical ways to mana*ge* symptoms, treatment demands, caregiving tasks and illness‐related uncertainty.

Higher hope may also be associated with more constructive dyadic coping behaviours. Patients with higher hope may be more willing to communicate their needs, accept support, participate in treatment‐related decisions and collaborate with their spouses in managing illness‐related challenges [48]. Similarly, spouses with higher hope may be more able to maintain supportive communication, share problem‐solving responsibilities and remain engaged in caregiving under treatment‐related stress [48]. These findings suggest that hope may be relevant not only to an individual’s psychological adjustment but also to their capacity to cope collaboratively with their partner [13, 14, 44].

Significant partner associations were also identified. Patient hope was positively associated with spouse dyadic coping, while spouse hope was positively associated with patient dyadic coping. The above findings support Hypothesis 2, suggesting that an individual’s hope is associated not only with their own coping strategies but also with their partner’s coping behaviours within the dyadic relationship.

Notably, one confidence interval was close to zero; therefore, this partner association should be interpreted with caution and confirmed in longitudinal research. This result builds upon previous studies, which have largely focused on hope or coping at the individual level for patients and spouses in isolation [34]. By applying the APIM, the present study highlights the potential interpersonal relevance of hope in couples coping with HCC after TACE [16]. The partner associations may be interpreted in light of Bodenmann’s systemic‐transactional model of dyadic coping, which proposes that stress experienced by one partner affects the other and that couples manage stress through communication, mutual support, joint problem‐solving and shared emotional regulation [13, 14]. A hopeful patient may communicate more openly, remain engaged in treatment and participate actively in shared coping efforts, which may be associated with reduced caregiving uncertainty and better dyadic coping in the spouse. Conversely, a hopeful spouse may provide reassurance, practical support and confidence in the family’s capacity to manage illness‐related challenges, which may be associated with more effective dyadic coping in the patient.

Taken together, Snyder’s hope theory and Bodenmann’s dyadic coping framework provide a useful explanation for the present findings. Hope may function not only as an individual psychological resource but also as a relational resource associated with how patients and spouses communicate, support one another and manage illness‐related stress together. Nevertheless, these findings should be interpreted cautiously because of the cross‐sectional design. The temporal direction of the associations cannot be established; effective dyadic coping may also contribute to the maintenance or enhancement of hope. Longitudinal studies are needed to examine the reciprocal relationships between hope and dyadic coping over time.

### 4.4. Implications for Nursing Management and Practice

The present findings have important implications for nursing management and clinical supportive care for patients with HCC after TACE and their spouses. The significant actor and partner associations indicate that hope and dyadic coping should not be assessed or addressed solely at the individual patient level. Although oncology care has traditionally focused on patients’ physical symptoms, treatment adherence and emotional distress, the present findings suggest that spouses’ hope and coping resources are also relevant to patients’ adjustment. Therefore, nursing care should recognise patients and spouses as an interdependent dyad and incorporate both partners into psychosocial assessment and supportive care planning.

First, ward nurses could incorporate brief assessments of hope and dyadic coping into routine predischarge assessment and follow‐up procedures for both members of the dyad, rather than assessing only the patient’s psychological status. Screening may be particularly important for couples in which either partner reports low hope, limited shared coping, communication difficulties, high caregiving strain or substantial distress. Early identification of such high‐risk couples can alert nurses to offer timely assistance or refer them to psychological services, social work support and palliative care.

Second, nurse managers may develop or organise couple‐oriented psychoeducation modules during hospitalisation for TACE. These modules should educate patients and their families about HCC, TACE‐related symptoms, treatment options and available health resources. They should also incorporate joint stress management training and problem‐solving sessions and teach participants how to establish realistic individual and family goals. Consistent with Snyder’s hope theory, nurses can assist patients and their families in cultivating both agency thinking and pathway thinking. Agency‐oriented support encourages each family member to persist and maintain a sense of control over their lives despite illness, whereas pathway‐oriented support helps couples select from various coping strategies—for example, drawing on family support networks or seeking professional medical advice. For patients with primary liver cancer, hope may be compromised by physical symptoms, uncertainty, treatment burden, financial constraints and limited perceived social support [21]. Consequently, hope‐based nursing support should be integrated with practical symptom management, emotional care, caregiver support and clear communication regarding treatment and follow‐up planning.

Third, the nursing team will create an extended follow‐up plan for the dyads at high psychosocial risk. A number of modes can be used for follow‐up monitoring of hope, dyadic coping, caregiver burden and new support needs after discharge and during repeated TACE cycles, such as telephone follow‐up, outpatient counselling, nurse‐led clinics and digital health platforms. Therefore, the above proactive follow‐up measures are required because HCC and its treatment frequently involve repeated hospitalisations, changes in symptoms and poor prognoses, which place continuous psychological and caregiving pressure on both patients and their spouses.

When the patient agrees, the spouse can also be added to the follow‐up consultation list to help identify any deficiencies in care that may have been missed during an assessment of the patient alone.

At the organisation level, these couple‐centred approaches could be added to the standard TACE nursing pathway. The nursing manager will establish a special two‐person team for assessment and referral, involve spouses in patient education and discharge planning and provide training on family‐oriented communication, caregiver assessment, hope‐focused support and psychosocial referral. Collaboration among oncology nurses, physicians, psychologists, social workers and palliative care professionals may further strengthen the continuity and comprehensiveness of support provided to patients and their spouses. Rather than replacing individualised care, a dyad‐centred approach extends it by recognising that the patient’s adjustment and the spouse’s coping are interconnected.

Overall, the findings support a shift from a predominantly patient‐focused model towards a more systemic and family‐centred approach to TACE nursing care. The observed interdependence between hope and dyadic coping suggests that supportive care strategies may be more responsive to the lived experience of HCC families when they address the psychological and relational needs of both partners. These findings provide a rationale for the future development and evaluation of hope‐centred, dyad‐based nursing support programmes for patients with HCC after TACE and their spouses.

## 5. Limitations

Several limitations should be acknowledged. First, the single‐centre design and relatively modest sample size may limit the generalisability of the findings to patients with HCC undergoing TACE and their spouses in other healthcare settings. Second, because this was a cross‐sectional study conducted after TACE, pretreatment levels of hope and dyadic coping, as well as their changes over time, could not be assessed. Therefore, the temporal ordering and causality of the observed actor and partner associations cannot be established. Hope and dyadic coping may change as patients and spouses experience variations in disease status, treatment response, symptoms, caregiving demands and family roles. Third, although some variables departed from normality, we used parametric tests given the relatively large sample size and their robustness to moderate violations of normality. The results should be interpreted with this limitation in mind. Future multicentre studies with adequately powered samples should use longitudinal designs with repeated assessments at clinically meaningful time points, including before TACE, during the early posttreatment period and at follow‐up. Such studies could validate the present findings and clarify the trajectories and reciprocal relationships between hope and dyadic coping throughout the illness course.

## 6. Conclusion

In conclusion, dyadic coping among patients with HCC following TACE and their spouses may require further support. Hope was significantly associated with dyadic coping at both the actor and partner levels, suggesting that hope may represent a potentially relevant focus for dyad‐centred nursing care. Healthcare professionals should consider moving beyond individual‐focused psychological support and recognise patients and their spouses as an interdependent dyad when planning and delivering supportive care. Nurse managers and nurse leaders may facilitate the implementation of dyad‐centred care by supporting assessment, education and referral pathways that address the needs of both patients and spouses. The findings provide preliminary evidence to inform the development of hope‐centred dyadic nursing interventions. However, longitudinal and intervention studies are needed to establish the temporal relationships between hope and dyadic coping and to evaluate the feasibility and effectiveness of such interventions.

## Author Contributions

Yu Wei, Yuan Liu and Ailing Hu were involved in the study design. Yu Wei, Yan Li and Lei Sun were involved in data collection. Yu Wei, Xiaofang Long and Ying Yin were involved in data analysis. Yu Wei and Yuan Liu were involved in manuscript preparation. Yuan Liu and Ailing Hu were involved in manuscript revision. Yu Wei, Xiaofang Long and Ying Yin contributed equally to this work.

## Funding

This research received no specific grant from any funding agency in the public, commercial or not‐for‐profit sectors.

## Conflicts of Interest

The authors declare no conflicts of interest.

## Supporting Information

Additional supporting information can be found online in the Supporting Information section.

## Supporting information


**Supporting Information** Supporting File S1: STROBE checklist for cross‐sectional studies, outlining the reporting items addressed in this manuscript.

## Data Availability

The data that support the findings of this study are available from the corresponding authors upon reasonable request.
