# Supplementary material for: Couple‐Level Coping and Hope Interdependence in Hepatocellular Carcinoma After Transcatheter Arterial Chemoembolisation: Insights From an Actor–Partner Interdependence Model
Source: J Nurs Manag. 2026 Sep 27;2026:3877436. doi: 10.1155/jonm/3877436 (PMC13617309; doi:10.1155/jonm/3877436)
Supplement: Supplementary file 1 [file JONM-2026-3877436-s001.zip › STROBE_Checklist_3877436.doc]

**STROBE Checklist – Cross-Sectional Studies**

**Manuscript ID:** 3877436

**Title:** Couple-Level Coping and Hope Interdependence in Hepatocellular Carcinoma after Transcatheter Arterial Chemoembolisation: Insights from an Actor–Partner Interdependence Model

**Journal:** *Journal of Nursing Management*

|  | Item No | Recommendation | Page  No |
| --- | --- | --- | --- |
| **Title and abstract** | 1 | (*a*) Indicate the study’s design with a commonly used term in the title or the abstract | **p. 1** |
|  |  | (*b*) Provide in the abstract an informative and balanced summary of what was done and what was found | **pp. 1-2** |
| Introduction | | |  |
| Background/rationale | 2 | Explain the scientific background and rationale for the investigation being reported | **pp. 2-4** |
| Objectives | 3 | State specific objectives, including any prespecified hypotheses | **pp. 4-5** |
| Methods | | |  |
| Study design | 4 | Present key elements of study design early in the paper | **p. 5** |
| Setting | 5 | Describe the setting, locations, and relevant dates, including periods of recruitment, exposure, follow-up, and data collection | **p. 5** |
| Participants | 6 | Give the eligibility criteria, and the sources and methods of selection of participants | **p. 5** |
| Variables | 7 | Clearly define all outcomes, exposures, predictors, potential confounders, and effect modifiers. Give diagnostic criteria, if applicable | **p. 6** |
| Data sources/ measurement | 8 | For each variable of interest, give sources of data and details of methods of assessment (measurement). Describe comparability of assessment methods if there is more than one group | **pp.6-7** |
| Bias | 9 | Describe any efforts to address potential sources of bias | **pp. 7-8** |
| Study size | 10 | Explain how the study size was arrived at | **p. 5** |
| Quantitative variables | 11 | Explain how quantitative variables were handled in the analyses. If applicable, describe which groupings were chosen and why | **pp.7-8** |
| Statistical methods | 12 | (*a*) Describe all statistical methods, including those used to control for confounding | **pp.7-8** |
|  |  | (*b*) Describe any methods used to examine subgroups and interactions | N/A |
|  |  | (*c*) Explain how missing data were addressed | **p.7** |
|  |  | (*d*) If applicable, describe analytical methods taking account of sampling strategy | N/A |
|  |  | (*e*) Describe any sensitivity analyses | N/A |
| Results | | |  |
| Participants | 13 | (a) Report numbers of individuals at each stage of study—eg numbers potentially eligible, examined for eligibility, confirmed eligible, included in the study, completing follow-up, and analysed | **p.7** |
|  |  | (b) Give reasons for non-participation at each stage | **p.7** |
|  |  | (c) Consider use of a flow diagram | N/A |
| Descriptive data | 14 | (a) Give characteristics of study participants (eg demographic, clinical, social) and information on exposures and potential confounders | **p.8** |
|  |  | (b) Indicate number of participants with missing data for each variable of interest | **p.7** |
| Outcome data | 15 | Report numbers of outcome events or summary measures | **pp.9-10** |
| Main results | 16 | (*a*) Give unadjusted estimates and, if applicable, confounder-adjusted estimates and their precision (eg, 95% confidence interval). Make clear which confounders were adjusted for and why they were included | **pp. 10–11** |
|  |  | (*b*) Report category boundaries when continuous variables were categorized | N/A |
|  |  | (*c*) If relevant, consider translating estimates of relative risk into absolute risk for a meaningful time period | N/A |
| Other analyses | 17 | Report other analyses done—eg analyses of subgroups and interactions, and sensitivity analyses | N/A |
| Discussion | | |  |
| Key results | 18 | Summarise key results with reference to study objectives | **pp.11-13** |
| Limitations | 19 | Discuss limitations of the study, taking into account sources of potential bias or imprecision. Discuss both direction and magnitude of any potential bias | **p.15** |
| Interpretation | 20 | Give a cautious overall interpretation of results considering objectives, limitations, multiplicity of analyses, results from similar studies, and other relevant evidence | **pp.11-15** |
| Generalisability | 21 | Discuss the generalisability (external validity) of the study results | **p.15-16** |
| Other information | | |  |
| Funding | 22 | Give the source of funding and the role of the funders for the present study and, if applicable, for the original study on which the present article is based | **p. 16** |
